# Supplementary figures and images for: Serum miR-192-5p levels predict the efficacy of pegylated interferon therapy for chronic hepatitis B
Source: PLoS One. 2022 Feb 14;17(2):e0263844. doi: 10.1371/journal.pone.0263844 (PMC8843190; doi:10.1371/journal.pone.0263844)

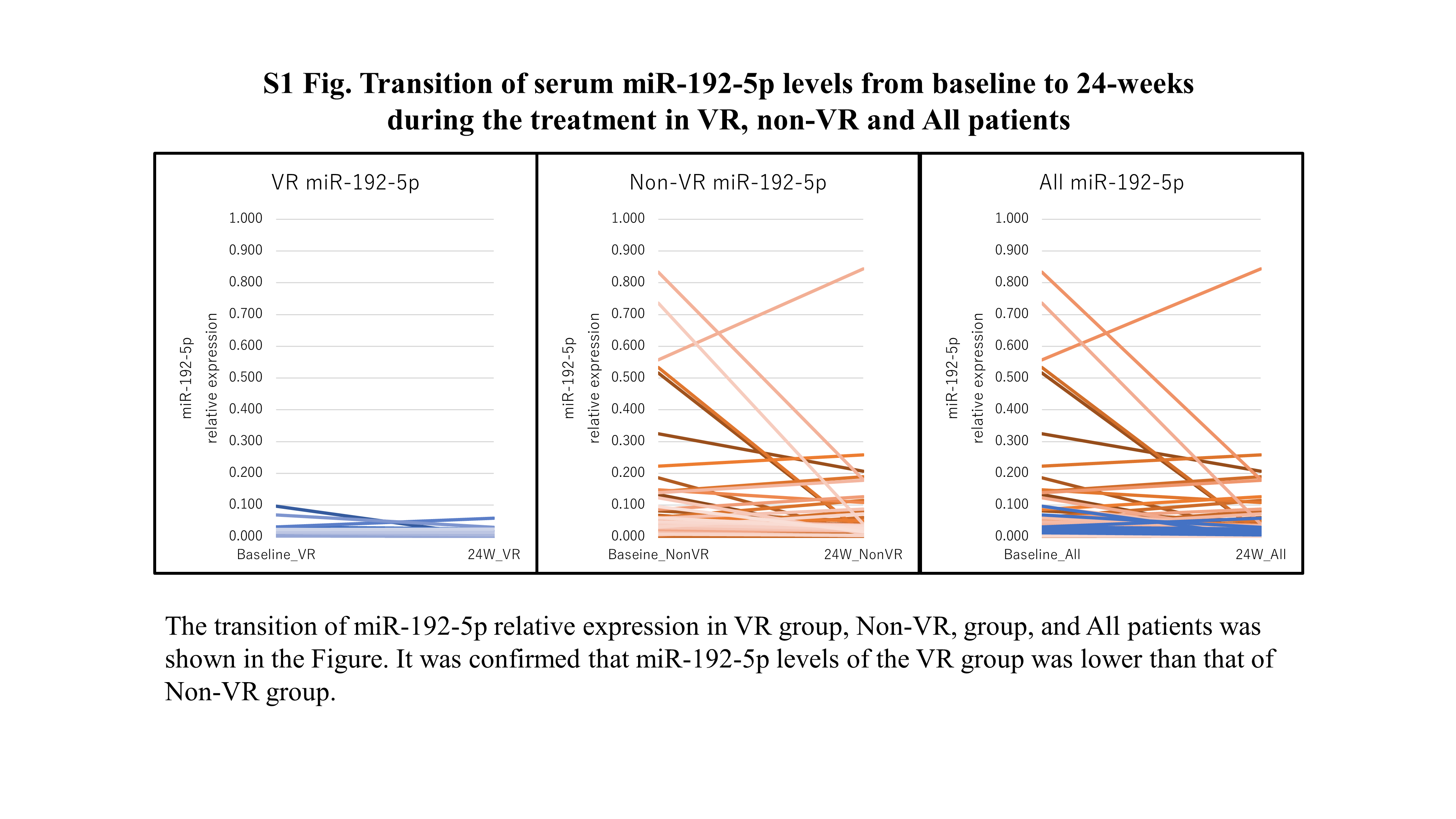

Supplement: S1 Fig — (TIF) [file pone.0263844.s001.tif]

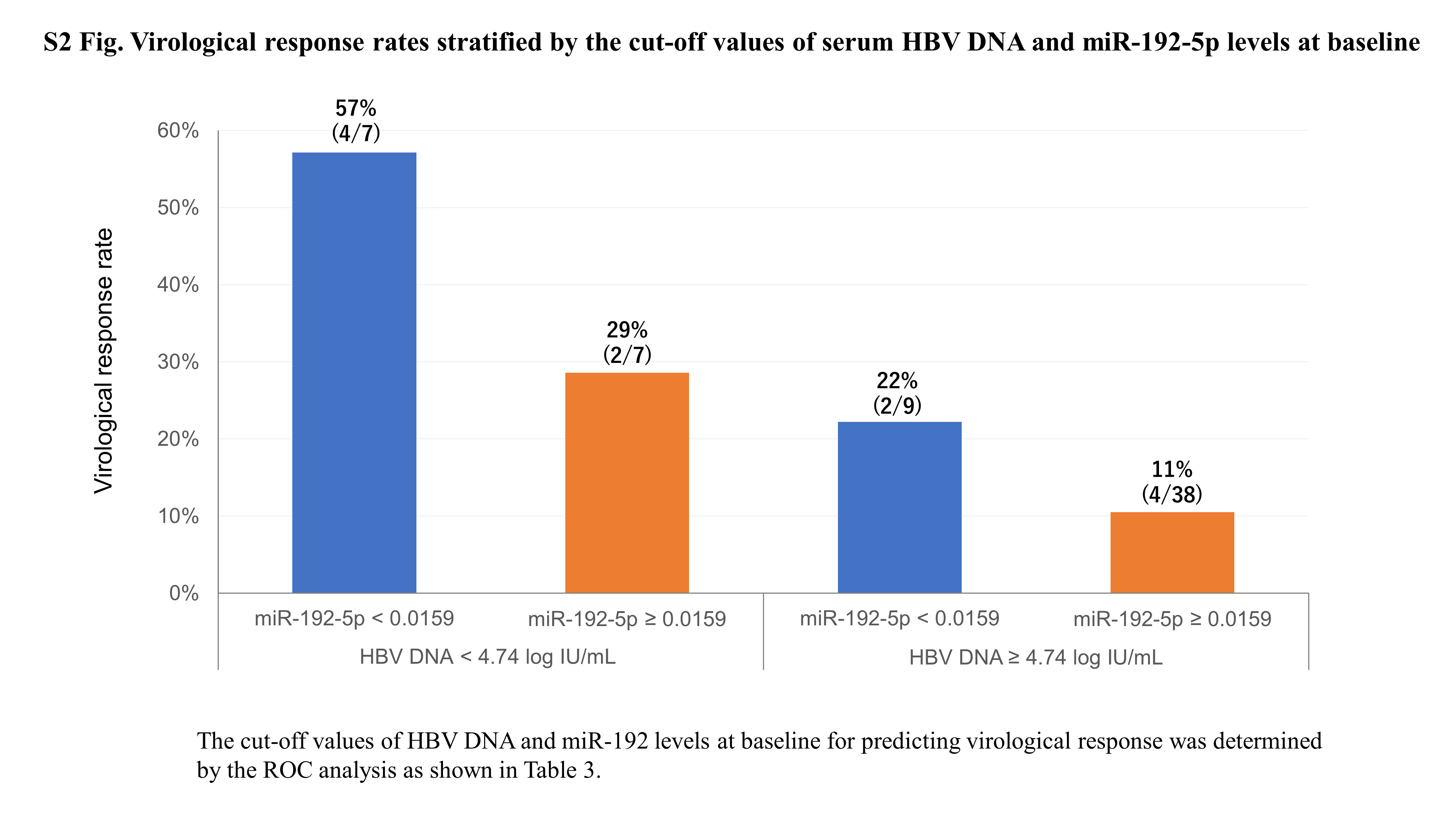

Supplement: S2 Fig — (TIF) [file pone.0263844.s002.tif]
