# Supplementary material for: Serum miR-192-5p levels predict the efficacy of pegylated interferon therapy for chronic hepatitis B
Source: PLoS One. 2022 Feb 14;17(2):e0263844. doi: 10.1371/journal.pone.0263844 (PMC8843190; doi:10.1371/journal.pone.0263844)
Supplement: S1 Table — (DOCX) [file pone.0263844.s003.docx]

S1 Table. Comparison of clinical characteristics of HBeAg-negative patients between VR and non-VR groups.

|  | Factor at baseline | | | Factor at 24 weeks | | | |
| --- | --- | --- | --- | --- | --- | --- | --- |
| Factor | HBeAg-negative VR (n = 9) | HBeAg-negative non-VR (n = 19) | *P* value | | HBeAg-negative VR (n = 9) | HBeAg-negative non-VR (n = 19) | *P* value |
| Age, years | 43 (34-46) | 41 (35-45) | 0.571 | | N.A. | N.A. | N.A. |
| Male, n (%) | 4 (44) | 10 (53) | 1.000 | | N.A. | N.A. | N.A. |
| HBV genotype A/B/C | 2 / 1 / 6 | 2 / 3 / 14 | 0.698 | | N.A. | N.A. | N.A. |
| AST (U/L) | 25 (22-63) | 32 (24-55) | 0.338 | | 35 (28-47) | 33 (27-56) | 0.961 |
| ALT (U/L) | 30 (26-126) | 47 (35-85) | 0.389 | | 32 (22-52) | 43 (26-72) | 0.731 |
| Platelet counts (×10^9^/L) | 216 (193-220) | 202 (176-230) | 0.694 | | N.A. | N.A. | N.A. |
| HBcrAg (log U/ml) | 3.6 (2.9-4.4) | 4.3 (3.8-4.7) | 0.325 | | 3.3 (2.9-4.1) | 3.2 (2.9-3.8) | 0.711 |
| HBsAg (IU/ml) | 3388 (202-8886) | 3070 (1698-10905) | 0.658 | | 1601 (37-2800) | 1837 (732-3882) | 0.488 |
| HBV DNA (log IU/ml) | 3.9 (3.5-5.7) | 5.0 (4.4-5.5) | 0.257 | | < 1.2 (ND-1.2) | 1.5 (< 1.2-2.2) | 0.115 |
| miR-192-5p | 0.014 (0.005-0.032) | 0.029 (0.018-0.054) | 0.055 | | 0.008 (0.006-0.025) | 0.024(0.017-0.035) | 0.094 |
| miR-320a | 0.178 (0.094-0.201) | 0.249 (0.147-0.285) | 0.069 | | 0.102 (0.091-0.256) | 0.200 (0.147-0.245) | 0.201 |
| miR-122-3p | 0.003 (< 0.001-0.007) | 0.001 (0.001-0.002) | 0.730 | | 0.001 (< 0.001-0.001) | 0.001 (< 0.001-0.003) | 0.605 |
| miR-6126-5p | 0.049 (0.029-0.161) | 0.155 (0.059-0.200) | 0.201 | | 0.044 (0.017-0.101) | 0.066 (0.020-0.108) | 0.461 |

Positive result (signal) below the quantitative HBV DNA concentrations was described as “<1.2”, and negative signal was described as “ND”.

Abbreviations: HBeAg, hepatitis B e antigen; VR, virological response; HBV, hepatitis B virus; AST, aspartate transaminase; ALT, alanine transaminase; HBcrAg, hepatitis B core-related antigen; HBsAg, hepatitis B surface antigen; miR, microRNA; ND, not detected; N.A., not available.
