# Supplementary material for: Serum miR-192-5p levels predict the efficacy of pegylated interferon therapy for chronic hepatitis B
Source: PLoS One. 2022 Feb 14;17(2):e0263844. doi: 10.1371/journal.pone.0263844 (PMC8843190; doi:10.1371/journal.pone.0263844)
Supplement: S2 Table — (DOCX) [file pone.0263844.s004.docx]

S2 Table. Comparison of clinical characteristics of patients between HBeAg-positive and HBeAg-negative.

|  | Factor at baseline | | |
| --- | --- | --- | --- |
| Factor | HBeAg-positive (n = 33) | HBeAg-negative (n = 28) | *P* value |
| Age, years | 32 (29-36) | 41 (35-46) | < 0.001 |
| Male, n (%) | 21 (64) | 14 (50) | 0.416 |
| HBV genotype A/B/C | 0 / 2 / 31 | 4 / 4 / 20 | 0.036 |
| AST (U/L) | 67 (38-110) | 31 (23-58) | 0.005 |
| ALT (U/L) | 107 (52-182) | 39 (27-95) | 0.002 |
| Platelet counts (×10^9^/L) | 192 (163-220) | 206 (178-229) | 0.183 |
| HBcrAg (log U/ml) | 6.9 (6.5-7.1) | 4.0 (3.4-4.6) | < 0.001 |
| HBsAg (IU/ml) | 15600 (4844-31296) | 3229 (1529-10687) | < 0.001 |
| HBV DNA (log IU/ml) | 7.9 (6.8-8.3) | 4.9 (3.8-5.7) | < 0.001 |
| VR, n (%) | 3 (9) | 9 (32) | 0.053 |

Abbreviations: HBeAg, hepatitis B e antigen; VR, virological response; HBV, hepatitis B virus; AST, aspartate transaminase; ALT, alanine transaminase; HBcrAg, hepatitis B core-related antigen; HBsAg, hepatitis B surface antigen; VR, virological response.
