# Supplementary material for: Serum miR-192-5p levels predict the efficacy of pegylated interferon therapy for chronic hepatitis B
Source: PLoS One. 2022 Feb 14;17(2):e0263844. doi: 10.1371/journal.pone.0263844 (PMC8843190; doi:10.1371/journal.pone.0263844)
Supplement: Attachment — Submitted filename: Response to Reviewers.docx [file pone.0263844.s006.docx]

**Reviewer #1**

**Comment 1:**

HBV-DNA is a routine biomarker to evaluate the efficacy of Peg-IFN for CHB. For this reason, even if authors clear that serum miR-192-5p is a biomarker is meaningless. The author should clear whether the serum miR-192-5p can replace HBV-DNA or help HBV-DNA to improve the prediction ability for the efficacy of Peg-IFN. Therefore, authors should add fresh evidence to illustrate serum miR-192-5p is better than HBV-DNA in predicting for the efficacy of Peg-IFN, or combine miR-192-5p and HBV-DNA could improve the prediction ability than single HBV-DNA, not for miR-192-5p.

**Response:**

Thank you for the important comment. As the reviewer pointed out, HBV DNA is a useful and reliable marker to predict the efficacy of Peg-IFN therapy for CHB. Actually, the present study showed that the AUC value of HBV DNA at baseline was the best. Therefore, serum miR-192-5p level cannot replace HBV DNA, but improve to predict the response to Peg-IFN therapy for CHB. As the reviewer suggested in the comment 3, we added data of accuracy using the cut-off values of HBV DNA and miR-192-5p levels in Table 4, which indicate the combination of HBV DNA and miR-192-5p levels improved accuracy as well as PPV and specificity. In addition, when we stratified the patients according to the cut-off values of serum HBV DNA and miR-192-5p levels at baseline, the VR rates were 57% in patients with HBV DNA < 4.74 and miR-192-5p < 0.0159; 29% in those with HBV DNA < 4.74 and miR-192-5p ≥ 0.0159; 22% in those with HBV DNA ≥ 4.74 and miR-192-5p < 0.0159; 11% in those with HBV DNA ≥ 4.74 and miR-192-5p ≥ 0.0159, respectively (S2 Fig). These data indicate that combining data of serum HBV DNA and miR-192-5p levels could improve the ability to predict the response to Peg-IFN therapy for CHB. We add these description in the text, in page 14-15, line 304-308, and in page 18, line 371-378.

**Comment 2:**

The area under the curve of HBV-DNA at base is 0.75, which is more than miR-192 at base (0.72) and miR-192 at 24-weeks (0.74). Those data are not indicative that miR-192-5p is a better biomarker than HBV-DNA for predicting VR. So, what is the significance purpose of this study?

**Response:**

As we described in the above response, serum miR-192-5p level could improve the ability to predict the response to Peg-IFN therapy for CHB. Furthermore, our results and previous findings let us speculate that miR-192-5p might be associate with HBV replication and antiviral immunity as we described in the discussion, in page 19, line 405-431. We added the significance of our study in the conclusion, in page 20, line 451-454. .

**Comment 3:**

In addition to the area under the curve, accuracy is another important index to evaluate diagnostic ability. Authors should add the accuracy of serum miR-192-5p and/or HBV-DNA for predicting VR.

**Response:**

Thank you for the suggestive comment. As we described in the response to the comment 1, we added the data of the accuracy of serum miR-192-5p and/or HBV-DNA for predicting VR in Table 4.
